# Media advocacy in catalyzing actions by decision-makers: case study of the advance family planning initiative in Kenya

**DOI:** 10.3389/fgwh.2023.1168297

**Published:** 2023-06-06

**Authors:** Irene Choge, Rammah Mwalimu, Sam Mulyanga, Sally Njiri, Beatrice Kwachi, Susan Ontiri

**Affiliations:** ^1^ Advance Family Planning Project, Jhpiego, Nairobi, Kenya; ^2^ International Center for Reproductive Health, Mombasa, Kenya

**Keywords:** media, advocacy, media advocacy, family planning, Kenya

## Abstract

Media can not only play a critical role in informing and educating the public on health issues, but it can make a powerful contribution to advocacy of public health matters. In Kenya, Advance Family Planning (AFP) initiative used this approach to further the country's progress in achieving family planning goals. This case study documents AFP experience in supporting media to engage leaders and decision-makers on the need to unlock bureaucratic bottlenecks that limit success of family planning services. AFP's media efforts added weight to the work of advocates who push for increased political commitments and investments in family planning. Media advocacy efforts helped catalyze actions by decision-makers across Kenya—focusing on strengthening accessibility and availability of contraceptive methods and fast-tracking implementation of policy actions to address adolescent pregnancy. Media advocacy efforts contributed to advancing family planning initiatives in the country. Media advocacy should be a key pillar of family planning programs and of other sectors.

## 1. Introduction

The media is considered a critical channel for information dissemination and communicating a broad range of health messages (1, 2). Public health programs have used broadcast media (radio and television), print media (newspapers), and social media to reach large audiences in awareness creation and behavior change communication campaigns (3–5). In addition to being a source of information, education, and entertainment, media can also be a channel for advocacy in advancing public health policy initiatives (6). Specifically, it can be used as an advocacy tool to help leaders set policy agendas, form discussions on various topical issues, and support perspectives that can shape the political decision-making process (7). But, the media in this regard is underutilized (6). Many individuals, including policymakers who are often the decision-makers, and public health programs do not appreciate the role that media has in shaping and improving public health programs and in holding officials accountable for their commitments. Moreover, media organizations often do not see themselves as contributors to public health, even though they play an important role in providing accurate, evidence-based, data-driven information on health topics (8). Media's role in advancing policy discourse is critical as oftentimes it can help drive citizens to pressure leaders to address health concerns (6).

In Kenya, the Ministry of Health typically uses broadcast and print media to widely convey health-related commitments and messages, including those around family planning (FP) (5). Many FP and social behavior change programs promote health messages on contraceptives and strive to dispel myths and misconceptions among communities (9–11). While a project may include an advocacy component in addition to core activities, e.g., service delivery or demand generation, there is limited information on how health programs, specifically FP, have used media for advocacy and accountability.

Advance Family Planning (AFP) implemented in Kenya by Jhpiego aims to increase the financial investment and political commitment needed to ensure access to quality (FP) through evidence-based advocacy. AFP project incorporated the use of media advocacy as a core component of its interventions in Kenya to complement AFP and stakeholders’ advocacy efforts by deploying media strategies that generate and sustain dialogue on the family planning needs in Kenya and beyond. The media created awarenes of family planning and the need for it among the people while challenging policy makers to play their role in helping improve access and acceptability of contraceptives. This paper highlights the lessons learned, challenges encountered, and recommendations for using media as an advocacy tool for FP programs in Kenya.

## 2. Program description

AFP was launched in 2009 to increase financial investments and political commitments to ensure access to voluntary, quality, FP through an evidence-based SMART advocacy (specific, measurable, attainable, relevant, and time-bound) approach (12). This approach is a guide to developing advocacy strategies that lead to quick wins by reaching the right decision-maker with the right message at the right time.

**Figure 1:**
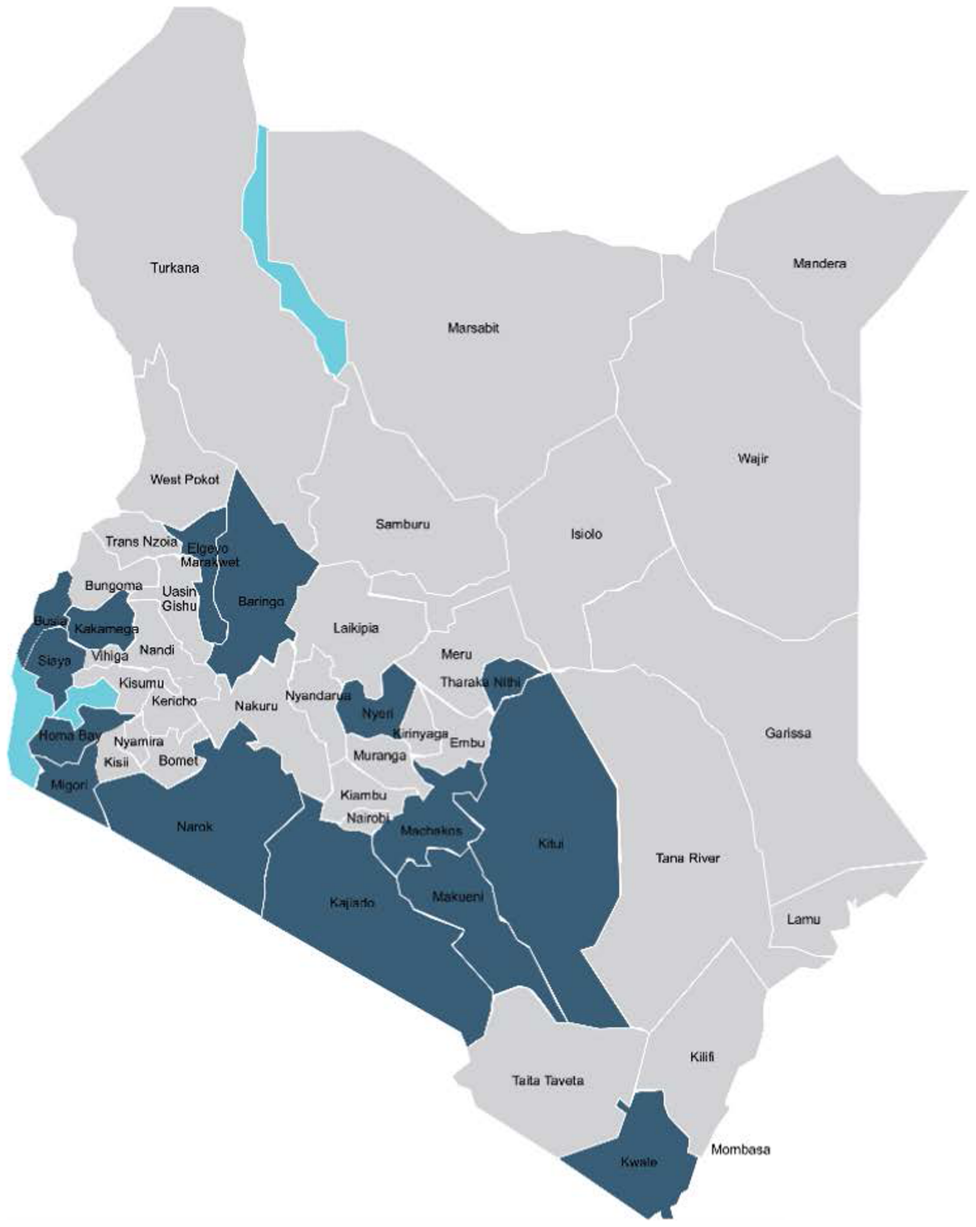
Kenya map showing AFP Media intervention counties.

In 2016, a media component was incorporated into the Kenya project to reinforce accountability on FP commitments. The project worked with journalists and editors from national and local media houses in 16 AFP focus counties (Kitui, Makueni, Kwale, Kakamega, Tharaka Nithi, Busia, Siaya, Homabay, Migori, Elgeyo Marakwet, Kisii, Nyeri, Narok, Kajiado, Baringo, and Nyamira) (Figure 1). The project focused on these counties because they had existing champions, political leadership, and had prioritized improving FP indicators in their county action plans.

### 2.1. Media advocacy engagement strategies

#### 2.1.1. Know your advocacy needs: create a distinct media strategy for advocacy

Media advocacy is defined as the strategic use of mass media to advance policy change (13). We developed a media advocacy strategy in 2016 to advance our overall FP advocacy plan. With the understanding that media advocacy is the strategic use of mass media for advocacy and centered on decision-makers to change policy, the strategy aimed to engage the media to produce data-driven, compelling FP stories and reports to catalyze decision-makers to act. The media strategy focused on engaging the media to overcome FP policy and implementation barriers; influence national and county governments, donors, and implementing partners to adopt or improve FP programs; profile evidence related to policy and programs under consideration or in practice; and refute misinformation. The strategy also focused on unlocking new domestic funding for programs, including private sector funds, highlighting progress towards FP commitments and plans, and supporting accountability.

The media strategy sought the production of newsworthy stories that would lead to “earned media,” in the form of donated public service announcements and additional news coverage, rather than paid media placements (14). To lead the media component, we recruited an award-winning journalist who was employed as part of the advocacy team. The health journalist supported journalists to access the latest data and research as well as training to improve their family planning storytelling. The project leveraged her expertise and understanding of how the media works to pitch FP as newsworthy content, leading to buy-in by media editors.

#### 2.1.2. Understand the media: conduct a media landscape assessment

In 2016, we conducted a media landscape assessment in Kenya to understand how different media houses work, identify the kind of stories they carry, and analyze how they frame issues. The assessment focused on reporting on FP to identify ways it could be improved. Terminologies related to FP were identified and used to review content from the major national print publications and radio and television stations.

The assessment team classified stories into feature stories (in-depth pieces) and event-related (news) stories to understand which newsrooms and reporters covered which type of health, reproductive health, or FP stories. The assessment provided insights into how different media houses treat stories about development and health, specifically reproductive health and FP. The assessment looked at story placement in newspapers and the amount of airtime given to FP stories. It also identified newsrooms with special segments on development, for potential FP stories. The assessors analyzed journalists’ style of reporting and became familiar with their work. They also conducted an analysis of newsroom editors and leadership to understand their positions on FP topics.

#### 2.1.3. Decide who to involve: select media houses and journalists

Based on the media landscape assessment, the project cultivated relationships with 16 media houses at the national and county levels. At the national level, we targeted organizations that have a wide, national reach and those with special development sections. At the county level, we focused on popular local radio stations.

AFP in Kenya organized one-on-one meetings with national and county-level editors and leadership, since they decide when or whether to carry a story. Our team explained how coverage of FP stories from different angles would help enrich their news coverage and discussed possible partnerships with the newsrooms in areas of access to reliable data; facilitating interviews with credible sources, including policymakers, FP users, and advocates and critics of FP; and capacity building for journalists. We secured the buy-in from editors who saw it as an opportunity to enrich their content with well-researched, in-depth, and compelling stories.

AFP requested the editors to select journalists for capacity-building workshops to institutionalize this engagement within the media houses. All the editors from the selected media houses responded positively, underscoring the importance of the assessment in establishing their interest. Editors chose journalists who were interested in reporting or writing feature stories on health, specifically reproductive health. At subnational level, selections were based on journalists’ interests in feature stories since many local radio stations do not have an expansive pool of beat reporters who specialize in health. Between 2016 and 2022, 41 journalists were identified from four national newspapers, four national TV stations, one subnational TV station and 18 radio stations (15 local stations, one national station and two international stations) (Table 1).

**Table 1:** Media outlets by channel engaged by AFP.

Coverage	Channel	Media stations
National Coverage	Radio	Citizen
	TV	NTV, Citizen, K24, KTN
	Print (newspaper)	The People Daily, The Standard, Nation, Star
Subnational Coverage	Radio	Inooro, Muuga, Ramogi, Mulembe, Milambo, Egesa, Athiani, Wikwatyo, County, Enee, Sidai, Chamgei, Kaya, Ranet, Kass
	TV	Ramogi
International Coverage	Online/Radio/TV	Deutsche Welle (DW), BBC

AFP Kenya staff and newsroom editors from the selected media houses who were willing to provide hands-on mentorship worked with the journalists and developed a training guide that combined three main tracks: storytelling, research data and the practice of Family Planning.

#### 2.1.4. Build capacity: develop and implement a capacity building strategy

Editors publish or kill a story based on the quality of an article or its lack of newsworthiness. Many health and development stories, especially FP stories, die because they may not have sufficient data or evidence to underpin their storytelling. We committed to help solve this problem by using workshops to build the capacity of journalists and by sharing more data and scientific evidence on family planning. With better access to data and research and armed with better storytelling skills, the journalists were able to produce numerous compelling stories that were published or aired on different media outlets. Between 2016 and 2022, we conducted 14 capacity building workshops for 41 journalists.

These full-day workshops ran for five days. The composition of participants varied, at times they were based on their outlets—radio, tv, and print and local vs national—other times there was a mix. Discussions, which also varied depending on current events, covered a wide spectrum of topics on health, science, and journalism, including reporting gaps and opportunities in health journalism. Participants learned how to localize a story if they were producing for a local audience and about story angles—how one idea could yield several pieces over time. They also learned how to use data in storytelling in order to make their stories complete as well as inspire decision-maker actions.

While participating journalists had radio, television, or print background, the workshops developed their multimedia storytelling skills and used practical exercises to look at how reporters can harness social media to tell and promote their stories. They also reviewed multimedia data resources and analytical tools. Journalists from national newspapers and television seemed acquainted with these skills, but journalists from the subnational level, which forms a large part of Kenya's pool of journalists, had not been exposed to social media storytelling tools.

During each workshop, journalists and their mentors went to the field to look for stories and then worked together during the production process. The workshops explored the concept of narrowcasting, as opposed to broadcasting, of directing FP and healthcare stories to the right channels and using different approaches for different audiences. The workshops also included story congresses, which provide journalists with an opportunity to share story ideas and get input from fellow journalists and editors on how to produce it.

#### 2.1.5. Monitor Implementation: conduct progress review to support the trained journalists

We developed a monitoring and evaluation plan that included indicators for tracking media advocacy initiatives. The project developed a story log template, which was completed by the trained journalists and included links to the articles, to track stories that had been aired by the journalists and actions taken because of the stories. Journalists who participated in the workshops shared logs of their stories, which the project used to determine which stories required follow-up. We created a WhatsApp mobile phone group to bring together the trained journalists, the mentors, and AFP staff. The journalists used the group to share ideas and asked for guidance. The mentors advised on approaches and other mentorship opportunities or collaborations.

From 2018–2022, we organized follow-up media workshops to evaluate and improve the capacity of participating journalists by assessing their progress in storytelling and offering additional mentorship.

#### 2.1.6. Thinking beyond the project: enhance sustainability

As part of its sustainability plan, we approached its pool of senior editor mentors to discuss ways in which they can continue mentoring journalists on a pro bono basis. They agreed to form an editorial mentorship board. The editorial mentorship board played a critical role in continuing virtual mentorship during the COVID-19 lockdown, when in-person review meetings were cancelled. With COVID-19 shifting attention to the pandemic, the board developed FP story ideas to help the journalists to put a spotlight on continuity of essential services during the pandemic.

## 3. Impact of media engagement

The media plays a critical role in setting agendas and framing issues. FP advocates have used stories produced by journalists to catalyze decision-makers to act and hold them accountable. To harness this unique role, we acknowledged the journalists’ independence; the project did not influence the story production process—all stories produced were led by journalists. Whereas AFP supported journalists, we did not demand any publicity from them, only encouraging them to produce the best stories on the cause. This allowed journalists to focus on telling the best possible stories they could. Cognizant of the fact that media houses are wary of promoting brands, our approach was to develop champions who can tell a story based on its newsworthiness without receiving incentives or paid advertisement. This freedom may have contributed to several advocacy wins as illustrated below.

• **Enhancing accountability:** In January 2019, three Migori-based journalists became aware of a stock-out of FP supplies in some of the facilities in Migori County and produced and aired a radio story covering the problem. Then, they aired an interactive radio talk show on FP with the county health promotion coordinator. During this show, women called in to concerns about contraceptive stockouts in some of the public health facilities. The county health promotion coordinator promised to act. Following the show, the county health department reviewed the status of FP stocks in all facilities and redistributed supplies to facilities experiencing stockouts. The county’s health promotion officer then issued a statement on radio about the redistribution and encouraged citizens to go to the facilities for services.
• **Catalyzing action on teenage pregnancy:** The 2014 Kenya Demographic and Health Survey revealed an adolescent pregnancy rate in Kwale County of 24%, which surpassed the national average of 18% (15). Trained journalists from national and county media platforms aired stories on adolescent pregnancy in Kwale County, including a documentary on NTV, a national TV station, which ran in September 2016; it won an international journalism award, the Michael Elliot Award; newspaper article segment on World Contraception Day, September 26, 2016; and weekly FP segments on Kwale’s local radio station (Radio Kaya) that showcased the gravity of the adolescent pregnancy situation in the county. Based on this increased visibility of the issues, in 2017, the Kwale County ministries of health, education, and youth and gender collaborated to develop a new costed action plan to address the growing concern of teenage pregnancy. The county went on to develop a multisectoral action plan to address teenage pregnancy. Following this media coverage, one of the county government health managers commented during a multisectoral teenage pregnancy working group meeting*,* “We saw [teenage pregnancy] as an issue, but the media coverage helped in raising the awareness of the community and other partners, who now want the matter addressed.”
• **Continuity of essential services during the COVID-19 pandemic:** In March 2020, the onset of COVID-19 pandemic led to government mitigation measures, including the abrupt interruption of FP services. In April 2020, FP uptake based on the health management information system service statistics data, dropped to the lowest level in 14 months (approximately 415,000 FP visits against an average of 433,000 over the previous 14 months). Many essential health services were halted while primary health care facilities only offered emergency services. The journalists under AFP, highlighted stories on how essential services, including FP, had been affected. Consequently, the reproductive health and FP stakeholders engaged with the Council of Governors to advocate for continuity of essential health services during the COVID-19 response. As a result, in May 2020, the Council of Governors Chairperson issued a memorandum to all 47 counties instructing them to provide essential health services, including FP, alongside COVID-19 response efforts. The memorandum emphasized the need to ensure the continued supply of FP commodities. The Ministry of Health reinforced the directive with specific guidelines for continuing essential services. In the months that followed, FP service provision not only recovered from its previous dip but also increased to levels higher than those seen in 2019 (an average of 470,000 FP visits).
• **Unlocking private sector money:** Public health programs utilize media for health education and communication and, in many instances, will pay for segments to be aired. Media houses consider these to be advertisements and a source of income. As part of its media advocacy strategy, we engaged senior editors and managers of popular county radio stations to provide airtime for FP stories at their own expense. This was achieved by packaging FP as a critical driver of community development and making a business case for the media. Between 2016 and 2021, 11 radio stations dedicated free airtime for FP stories, which would have cost $757,150, excluding production costs. Commitments from local media houses included airing feature stories that linked FP to development and following up the stories with live, call-in discussions by hosting experts in the studio. The free segments provided an opportunity for stakeholders to provide FP information, including dispelling myths and misconceptions, without cost.

### 3.1. Unanticipated outcome of media engagement

#### 3.1.1. Establishment of FP journalist community of practice

Training of journalists on their own has not been effective in the past due to lack of continuous engagement. Therefore, we set up a community of practice to bring together participating journalists and editors to provide a platform for continuous engagement, peer-to-peer learning, sharing of resources, and discussing challenges and opportunities. Due to the shared vision that was inculcated from the onset, journalists from competing media houses decided to collaborate to amplify FP stories nationwide. They formed a WhatsApp group for day-to-day interactions.

#### 3.1.2. Journalist-led FP stories

**Figure 2:**
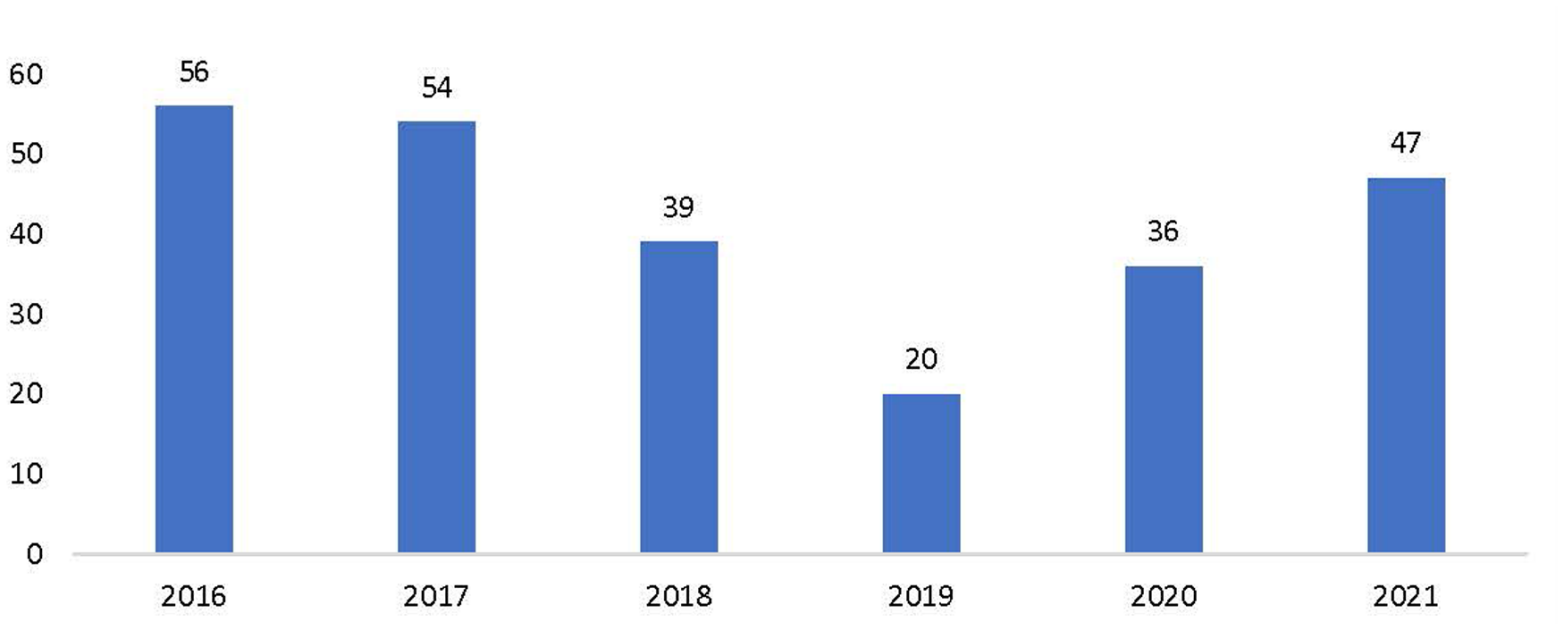
Number of FP advocacy stories aired or published by trained journalists.

The workshops had the corresponding benefits of introducing the process of creating a critical mass of FP storytellers in Kenya and “strengthening the program's relationship with partners in the country.” As a result of the first workshop, within the first two months, journalists published or aired 31 stories. Because these stories were journalist-led, no payment was made for the coverage, which is integral for sustainability. Continuous mentorship sustained momentum, leading to an increase in the number of relevant stories aired at various media outlets. By the end of 2021, more than 250 stories on FP had been aired or published (Figure 2). Additional workshops increased the pool of journalists who are interested in FP-related issues.

#### 3.1.3. Scale up to other countries

The media advocacy engagement strategy in Kenya proved to be scalable; as a result, AFP adopted this approach, scaling up implementation in Uganda, Nigeria, and Bangladesh.

## 4. Challenges

Although progress has been made since the start of the project in Kenya, FP is still not widely accepted, which remains a major challenge to increased media coverage. During performance review meetings, some trained journalists reported that they faced opposition from editors based on the editor's personal beliefs. This was especially noticeable among journalists who were from faith based media houses. Even though they had been identified to be trained, they did not receive adequate support needed to air their stories.

Another challenge was that some journalists did not consistently produce FP stories. FP stories are not commercial advertising, which generates income for the media house, which makes it difficult for journalists to have more engagement on FP since most of them are paid for the work that they deliver. Moreover, during the onset of COVID-19 pandemic, due to reduced revenues, media houses sacked six of the trained journalists, further reducing the pool of journalists airing FP-related stories.

## 5. Lessons learned

Following the five years of implementation of media advocacy, we concluded that the following lesson learned, and best practices are essential when conducting media advocacy.

• **Do not expect journalists to report a story from your perspective.** When we trained journalist, they had the freedom to write stories as they deemed fit. This at times did not go as per the project’s expectation. Although we invested in training and mentoring a journalist, we did not expect that they would report stories from the project’s angle. They exercise their own independence and thrive on pride that they are leading the story, not the project implementer. By adopting this approach, the journalists are able to own and drive the process and AFP was able to get a critical mass of FP stories.
• **Do not expect journalists to profile your organization.** An organization working in media advocacy must trade off its own visibility, public relations, for the public good. For greater impact, we decided to take a back-seat and allow the journalists to focus on issues and not profile Jhpiego as the implementing organization. Many organizations would like to be profiled to enhance their visibility, but this is often interpreted as public relations, which should be paid for, not as a public interest story. If a story is led by an implementing partner, decision-makers tend to look at issues such as who are the funders and what is their motivation; a credible story can be dismissed on the perceived ground of a vested interest. Moreover, paying for content for FP media advocacy may negate efforts since it might be misconstrued as pushing for a specific agenda. Thus, journalist-led stories, earned media, might provide gains that are sustainable beyond the life of an advocacy project.
• **Involve editors right from the onset to rally support.** Editors play a critical role in content production. Thus, it is important to engage the editors during development of training materials and give them an opportunity to co-facilitate sessions. Having a pool of trained journalists is not sufficient to guarantee expected outputs. A trained journalist can write a story, but the final decision to air or publish rests with the editors.
• **Help journalists to see how FP links other issues so they can continuously feature stories.** By linking FP stories with sustainable development goals and other aspects of development, journalists can sustain newsworthiness of FP and report stories from different angles. For instance, when there are restrictions of movement such as during emergencies, conflicts, or pandemics, trained journalists can capture human-interest stories that others are not focusing on, which can bring them to local and national attention. During the COVID-19 curfews and movement restrictions, journalists brought local and national attention to the need for continuity of essential health service at a time when FP programs and advocates were not able to raise the problem due to competition from other issues for the attention of decision-makers.
• **Instill social media as a tool for airing stories and use of media platforms as an influence.** With the onset of COVID-19 pandemic, some trained journalists lost their jobs. However, they continued their conversation through their social media presence due to their massive following.

## 6. Conclusion

The Advance Family Planning (AFP) worked to achieve the global goal of expanding access to quality contraceptive information, services, and supplies by providing decision makers with evidence that family planning is a sound investment for individuals, communities, and a nation’s future, and is a fundamental part of universal health coverage and achieving the Sustainable Development Goals. This paper offers AFP project's perspective on implementation of advocacy work in Kenya that incorporated media strategy as a core component of advocacy for engaging FP decision-makers. Our experience shows that media can play an important role in prompting decision-makers to act. It can also hold them accountable for their actions (or lack of action). For example, following the publication of a story titled ‘Policy stalemate: To offer contraceptives to adolescents or not?, in addition to other advocacy efforts, MoH resumed consultation convening on draft reproductive health strategy. Media advocacy efforts also contributed to the fast tracking of Kwale County’s multisectoral action plan on addressing teenage pregnancy. The multisectoral approach to addressing teenage pregnancy was later scaled up to 7 other counties and led to a national dialogue on the burden of teenage pregnancy. On advocacy efforts relating to expanding access to all contraception services, the example of Migori county redistributing FP commodities to facilities that lacked certain methods shows how the media helped in challenging the county governments to meet their commitments to the citizens and ensure delivery of services to the last mile.

WHO estimates that family planning increases economic growth per capita by 20-30% and avert 54% of preventable maternal deaths and 47% of preventable child deaths. With such great returns in investing in FP, advocacy is essential if countries are to meet their global commitments on FP of delivering contraceptives, information and services and upholding human rights. The media can spotlight and amplify community voices that are ignored during policy dialogues and ensure follow-up by policy and program leaders.

## Data Availability

The original contributions presented in the study are included in the article, further inquiries can be directed to the corresponding author.
